# Tamoxifen Decreases Lithium-Induced Natriuresis in Rats With Nephrogenic Diabetes Insipidus

**DOI:** 10.3389/fphys.2018.00903

**Published:** 2018-07-12

**Authors:** Stine Julie Tingskov, Tae-Hwan Kwon, Jørgen Frøkiær, Rikke Nørregaard

**Affiliations:** ^1^Department of Clinical Medicine, Aarhus University, Aarhus, Denmark; ^2^Department of Biochemistry and Cell Biology, School of Medicine, Kyungpook National University, Daegu, South Korea

**Keywords:** tamoxifen, lithium, natriuresis, ENaC, sodium transporter

## Abstract

Lithium is widely used in the treatment of bipolar affective disorders, but often causes nephrogenic diabetes insipidus (NDI), a condition characterized by a severe urinary concentrating defect. Lithium-induced NDI is associated with dysregulation of the amiloride-sensitive epithelial sodium channel (ENaC), which is essential for renal sodium reabsorption. Sex hormones have been shown to affect the expression of aquaporin-2 (AQP2) and sodium transporters. Therefore, we evaluated whether tamoxifen (TAM), a selective estrogen receptor modulator (SERM), would affect lithium-induced dysregulation of ENaC subunits and natriuresis. Rats were fed with lithium-containing food for 2 weeks to induce NDI and natriuresis. TAM was administered daily via gastric gavage after 1 week of lithium administration. Lithium treatment alone resulted in increased urinary sodium excretion and significant reduction of βENaC and γENaC at both RNA and protein levels. In addition, the plasma sodium level reduced after lithium treatment. Administration of TAM prevented increased urinary sodium excretion as well as attenuated the downregulation of βENaC and γENaC. Consistent with these findings, immunohistochemistry (IHC) showed stronger labeling of βENaC and γENaC subunits in the apical domain of the collecting duct cells in the cortical tissue of lithium-fed rats treated with TAM. Other major sodium transporters including NaPi-2, NKCC2, Na/K-ATPase, and NHE3, are believed not to have an effect on the increased urinary sodium excretion since their expression increased or was unchanged after treatment with lithium. In conclusion, the results demonstrated that TAM rescued the adverse effects of the lithium-induced increase in fractional excretion of sodium after the establishment of lithium-induced NDI.

## Introduction

Lithium is an important drug, which is widely used in the management of bipolar disorders. It has been shown to cause nephrogenic diabetes insipidus (NDI) in up to 40% of patients receiving lithium (Stone, 1999). Lithium-induced NDI is associated with polyuria caused by marked downregulation of aquaporin 2 (AQP2) and AQP3 proteins (Marples et al., 1995; Kwon et al., 2000; Nielsen et al., 2003).

Lithium treatment is also accompanied by increased urinary sodium excretion (Kwon et al., 2000; Nielsen et al., 2003; Zhang et al., 2015) and previous studies have demonstrated dysregulation of the amiloride-sensitive epithelial sodium channel (ENaC) in cases of lithium-induced NDI (Nielsen et al., 2003). ENaC is a heteromeric protein with three subunits α-, β-, and γENaC (Canessa et al., 1994) and is the main transport pathway for apical sodium reabsorption in the connecting tubules and the collecting ducts (Butterworth, 2010). The sodium reabsorption by ENaC is mediated by the hormones aldosterone and vasopressin and is associated with an alteration in the expression of the individual ENaC subunits (Garty and Palmer, 1997; Masilamani et al., 1999). Previous studies identified marked downregulation of the ENaC subunits β and γ in the cortical and outer medullary (OM) collecting duct in lithium-induced NDI (Nielsen et al., 2003). These changes, affecting the chief sites of sodium reabsorption in the collecting duct, indicate that this sodium channel is likely to play a role in lithium-induced natriuresis (Nielsen et al., 2003). Compatible with the distal effects of lithium, Kwon et al. showed that several major renal sodium transporters, including the type 3 Na/H exchanger (NHE3), Na-K-ATPase, and Na-K-2Cl cotransporter (NKCC2) were not downregulated in response to lithium treatment, (Kwon et al., 2000) suggesting that these sodium transporters are not likely to be involved in the development of natriuresis after lithium administration. Strategies to prevent natriuresis in lithium-induced NDI have so far been unsuccessful; therefore, future studies will search for alternative drug targets for the treatment of this disease.

Several studies have demonstrated an important role for estrogen in regulating water and salt balance (Brunette and Leclerc, 2001; Stachenfeld et al., 2003). Estrogen induces fluid retention, which is caused partly by increasing the tubular sodium reabsorption (Brunette and Leclerc, 2001). Tamoxifen (TAM) is a selective estrogen receptor modulator (SERM) that has an anti-estrogenic effect on the mammary glands and exhibits estrogenic effects on the cardiovascular and skeletal systems (Lonard and Smith, 2002; Kim et al., 2014). Importantly, we have recently demonstrated that TAM is able to attenuate the downregulation of AQP2 in rats with lithium-induced NDI (Tingskov et al., 2018). We showed that TAM rescued the adverse effects of lithium-induced polyuria after the establishment of lithium-induced NDI, and we now want to investigate whether TAM also plays a role the regulation of lithium-induced natriuresis after induction of lithium-NDI.

In this study, we hypothesized that TAM might have beneficial effects on increased sodium excretion-related NDI by attenuating lithium-induced natriuresis, along with maintenance of ENaC expression. The purpose of the present study was to investigate whether TAM regulates the expression of ENaC subunits and other sodium transporters or exchanges and whether TAM attenuates natriuresis and kaliuresis after lithium treatment.

## Materials and Methods

### Experimental Animals and Protocol

Experiments were performed on adult male Sprague-Dawley rats, initially weighing 198 ± 11.5 g. The rats were maintained in metabolic cages for collection of 24 h urine samples, with a 12:12 h light-dark cycle, a temperature of 21 ± 2°C, and humidity of 55 ± 2%. The rats had free access to standard rat chow (Altromin, Lage, Germany) and water during the experiment. In protocol 1 the effect of TAM was tested in health control rats for 7 days. TAM (Sigma-Aldrich Co. T5648) was mixed with corn oil and administered by daily oral gavage. The control group received corn oil by gavage once every day. One group of control rats was treated corn oil (*n* = 5) and one group was treated with TAM at a dose of 50 mg/kg for 7 days (*n* = 5). The rats were maintained in metabolic cages for collection of 24 h urine samples to be used for measuring urine output and sodium excretion. In protocol 2 the effect of TAM was tested in lithium treated rats. For lithium therapy, lithium chloride (Sigma-Aldrich, Copenhagen, Denmark) was added to the chow to achieve a concentration of 40 mmol/kg dry food. Lithium-treated rats received food containing 40 mmol/kg for 14 days (LiCl; *n* = 10), affording therapeutic levels of lithium in the serum (Tingskov et al., 2018). The rats used as controls were fed an unsupplemented standard food (Altromin, Lage, Germany) for 14 days (CTL; *n* = 8). Two groups of lithium-fed rats were treated with TAM at a dose of 25 mg/kg (LiCl + TAM 25; *n* = 10) and at a dose of 50 mg/kg for the last 7 days of the experimental period (LiCl + TAM 50, *n* = 12). TAM was mixed with corn oil and administered by daily oral gavage. The control and LiCl groups received corn oil by gavage once every day. Water consumption was monitored continuously, and the rats were maintained in metabolic cages for collection of 24 h urine samples to be used for clearance studies. After 14 days, the rats were sacrificed and the kidneys were removed and prepared for immunohistochemistry (IHC), quantitative PCR, and semi-quantitative immunoblotting.

All procedures were performed in accordance with the Danish national guidelines for the care and handling of experimental animals and in cooperation with a veterinarian. The animal protocols were approved by the Institute of Clinical Medicine, Aarhus University, according to the licenses for the use of experimental animals issued by the Danish Ministry of Justice. Animals has access to food and water *ad libitum*, were housed in a 12:12 h light-dark cycle, a temperature of 21 ± 2°C, and a humidity of 55 ± 2% and inspected daily. Animals were euthanized by cervical dislocation after the use of excess anestetics (Sevoflurane, Abbott Scandinavia, Sweden) according to institutional guidelines and approved protocols. These male rats have been used in our previous study and functional data after treatment with lithium and TAM have been published (Tingskov et al., 2018).

### Blood and Urine Chemistry

Before the rats were euthanized, blood samples were taken from the aortic bifurcation to determine plasma sodium levels. Plasma and urinary sodium and potassium levels were determined using a Roche Cobas 6000 analyzer (Roche Diagnostics, Risch-Rotkreuz, Switzerland). Lithium measurements were completed using Advia Chemistry XPT (Siemens Healthcare A/S, Ballerup, Denmark).

### RNA Extraction and Quantitative PCR

Total RNA was isolated from the kidney cortex and OM with a Nucleospin RNA II mini kit according to the manufacturer’s protocol (Macherey Nagel, Düren, Germany). RNA concentration was determined by spectrophotometry at 260 nm and the samples were then stored at −80°C. cDNA synthesis was performed using 0.5 μg of RNA with the AffinityScript QPCR cDNA synthesis kit (Life Technologies, Thermo Fisher Scientific, Cambridge, MA). For QPCR, 100 ng of cDNA served as the template for PCR amplification using the SYBR^®^ Green QPCR Master Mix according to the manufacturer’s instructions (Life Technologies) by using an Aria Mx3000P qPCR System (Agilent Technologies, Santa Clara, CA, United States). *GAPDH* was the control gene. The primer sequences were as follows: *αENaC*: sense 5′-TTCTGCACCAACACCACCAT-3′ and anti-sense 5′-GTTGAGGCTCACTGGGTAGC-3′; *βENaC*: sense 5′-CTGTGTCTTCCAGCCTGACA-3′ and anti-sense 5′-GCAGCCTCAGGGAGTCATAG-3′; *γENaC*: sense 5′-CTACCAGCAACACCCCAACT-3′ and anti-sense 5′-GCTACAGGATTGCTTGCACA-3′; and *GAPDH*: sense 5′-TAAAGGGCATCCTGGGCTACACT-3′ and anti-sense 5′-TTACTCCTTGGAGGCCATGTAGG-3′.

### Immunohistochemisty

For immunolabeling, the removed kidneys were fixed by retrograde perfusion in 4% paraformaldehyde in 0.01 M phosphate-buffered saline (PBS) buffer and washed in PBS. The fixed kidneys were then dehydrated in graded ethanol and left overnight in xylene. The tissue was embedded in paraffin and then cut into 2 μm-thick slices on a rotary microtome (Thermo Scientific, Microm HM 355S).

To evaluate the localization of α-, β-, and γ-ENaC, the tissue slices were exposed to immunoperoxidase labeling and endogenous peroxidase was blocked in 35% H_2_O_2_ dissolved in methanol. Afterward, the sections were boiled in TEG buffer for 10 min for antigen retrieval. Non-specific binding of immunoglobulins was prevented by incubating the sections in NH_4_Cl for 30 min followed by blocking in PBS containing 1% bovine serum albumin (BSA), 0.2% gelatin, and 0.05% saponin. The sections were incubated with primary antibodies diluted in 0.1% BSA and 0.3% Triton X-100 at 4°C overnight in a humidity chamber. After being rinsed in the PBS solution for 3 × 10 min, the sections for immunoperoxidase labeling were incubated with horseradish peroxidase (HRP)-conjugated secondary antibody for 1 h at room temperature. After rinsing, the sections were incubated in 3,3′-diaminobenzidine for 10 min in order to visualize the peroxidase and counterstained with Mayer’s hematoxylin. Conventional light microscopy was performed using an Olympus BX50 microscope and CellSens Imaging software (Olympus).

### Protein Isolation and Semi-Quantitative Immunoblotting

Renal cortical and OM tissues were homogenized in dissecting buffer (0.3 M sucrose, 25 mM imidazole, and 1 mM EDTA; pH 7.2) containing protease inhibitors (Phosphatase Inhibitor Cocktails 2 and 3, Sigma-Aldrich, St. Louis, MO, United States and Complete Mini Protease Inhibitor Cocktail Tablets, Roche, Hvidovre, Denmark). The tissue was homogenized for 240 s at 50 Hz by using a TissueLyser LT (Qiagen, Hilden, Germany) and then centrifuged at 4,500 × g at 4°C for 10 min. Gel samples were prepared from the supernatant in Laemmli sample buffer containing 2% SDS. The total protein concentration of the homogenate was measured using a Pierce BCA protein assay kit (Roche). The proteins were size-separated on 12% Criterion TGX Precast Gel and then electrotransferred to a nitrocellulose membrane. Afterward, the blots were blocked with 5% non-fat dry milk in PBS-T as described previously (Ostergaard et al., 2014). After washing with PBS-T, the blots were incubated with primary antibodies overnight at 4°C and visualized with HRP-conjugated secondary antibodies for 1 h at room temperature by using an enhanced chemiluminescence system (ECL, Amersham ECL Plus, GE Healthcare). All western blots were normalized to total protein, as measured using stain-free technology (Gürtler et al., 2013).

### Primary Antibodies

For western blot and immunofluorescence analyses of tissue sections, we used previously characterized specific antibodies. For western blot, the following antibodies were used: αENaC, βENaC, γENaC (Masilamani et al., 1999), NHE3 (ab95299, Abcam, Cambridge, United Kingdom), NaPi (LL696AP) (Biber et al., 1996; Kwon et al., 2000), NKCC2 (LL320AP) (Ecelbarger et al., 1996; Nielsen et al., 1998; Kim et al., 1999), and Na/K-ATPase (Millipore, 05-369). The following antibodies were used for IHC: βENaC (GTX41971, GeneTeX, Irvine, CA, United States) and γENaC (Masilamani et al., 1999). Horseradish-conjugated secondary antibodies were used (Life Technologies, Thermo Fisher Scientific, Cambridge, MA, United States). Furthermore, polyclonal goat anti-mouse IgG [P447] or polyclonal goat anti-rabbit IgG [P448] (Dako, Glostrup, Denmark) was used.

### Statistical Analysis

Data are presented as means ± SEM. Statistical comparison of two experimental groups was performed was performed using non-parametric Mann Whitney test. Multiple comparisons between several experimental groups were performed using a non-parametric one-way analysis of variance (ANOVA) followed by Tukey’s multiple-comparisons test. GraphPad Prism software (GraphPad Software, La Jolla, CA, United States) was used for all statistical analysis. *P*-values < 0.05 were considered significant.

## Results

### TAM Did Not Affect Urinary Sodium Excretion in Control Rats

**FIGURE 1:**
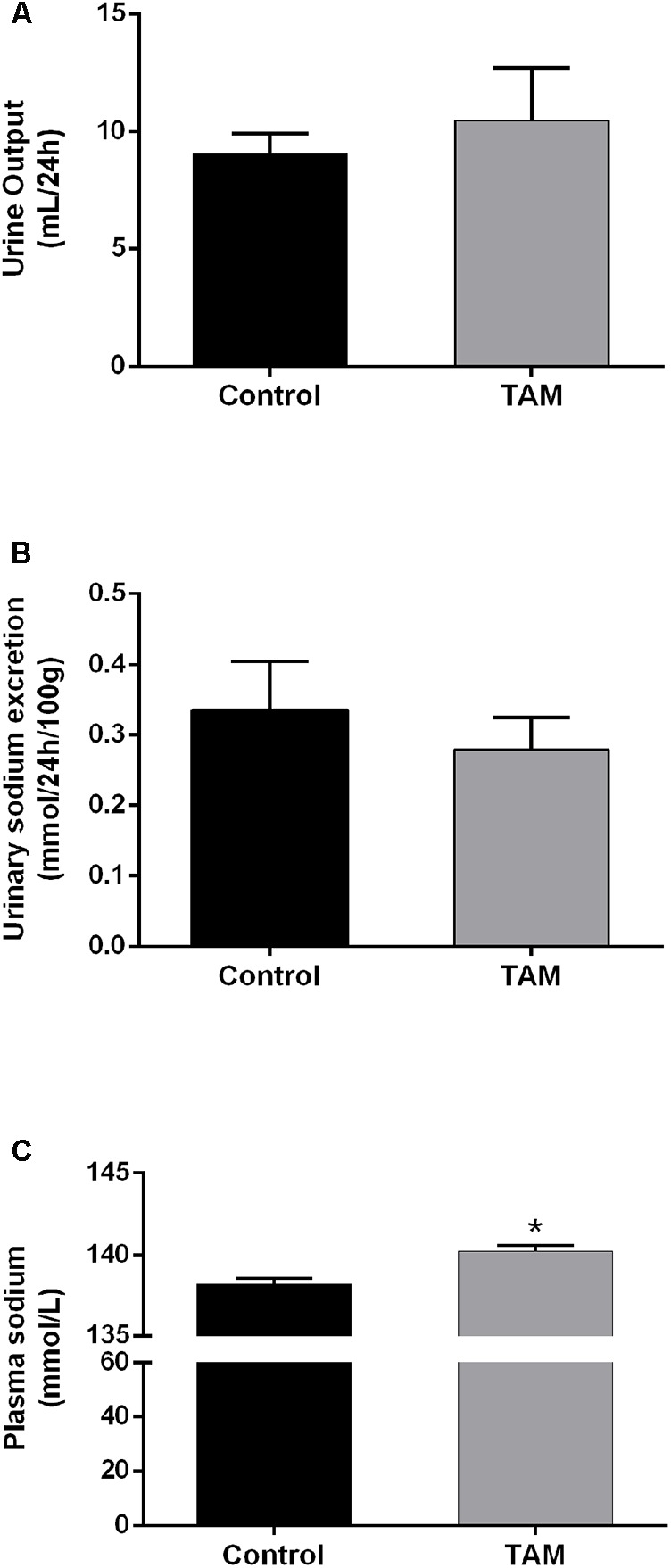
TAM did not affect sodium excretion in control rats. **(A)** Urine output in rats treated with TAM at day 7. **(B)** Urinary sodium excretion in rats treated with TAM at day 7. **(C)** Plasma sodium level at the end of the experiment. Each bar represents the mean ± SEM values; *n* = 5 for control rats and *n* = 5 for TAM treated rats. Comparisons between the experimental groups were performed using non-parametric Mann Whitney test. *P* < 0.05 was considered statistically significant indicated by ^∗^*P* < 0.05 compared to controls.

The effect of TAM on the urine output and sodium excretion was evaluated in normal control rats. Rats were treated with TAM (50 mg/kg) for 7 days and urine output as well as sodium excretion was analyzed. Our data showed that TAM treatment alone had no effect on urine output as well as urinary sodium excretion (**Figures 1A,B**). The plasma sodium level was slightly, but significantly, increased after TAM treatment, probable due to very little variation between the TAM treated and non-treated control rats (**Figure 1C**).

### TAM Improves Increased Urinary Sodium Excretion in Lithium-Treated Rats

**FIGURE 2:**
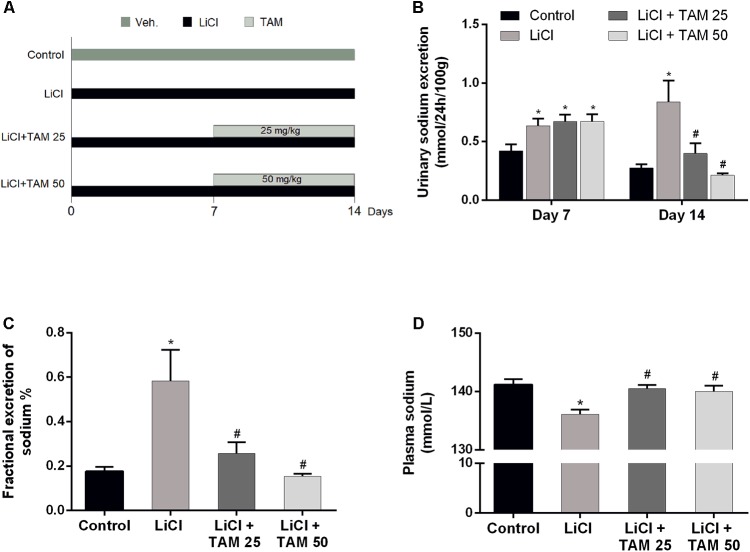
TAM improved increased urinary sodium excretion in the lithium-treated rats. **(A)** Schematic workflow for LiCl and TAM treatments. **(B)** Urinary sodium excretion in rats fed with normal or lithium-containing food, with and without TAM treatment at day 14. **(C)** Fractional excretion of sodium (%) on day 14. **(D)** Plasma sodium level at the end of the experiment. Each bar represents the mean ± SEM values; *n* = 8 for control, *n* = 10 for LiCl, *n* = 10 for LiCl + TAM 25, and *n* = 12 for LiCl + TAM 50 groups. Multiple comparisons between the experimental groups were performed using one-way ANOVA followed by using Tukey’s multiple-comparisons test. *P* < 0.05 was considered statistically significant indicated by ^∗^*P* < 0.05 compared to controls and ^#^*P* < 0.05 compared to LiCl-treated rats. TAM, tamoxifen; LiCl, lithium chloride.

To investigate whether TAM alleviates lithium-induced natriuresis and kaliuresis, three groups of rats were treated with lithium for 14 days. After 7 days, two groups of rats received an additional 1 week treatment with TAM at doses of 25 and 50 mg/kg (**Figure 2A**). All three groups of rats fed with lithium-containing food showed an increased urinary output as well as increased urinary sodium and potassium excretion at day 7 before TAM treatment was initiated (**Figure 2B** and **Table 1**). At day 14, urinary sodium excretion (**Figure 2B**) as well as fractional excretion of sodium (**Figure 2C**) remained increased in the LiCl-treated rats. TAM treatment significantly reversed the lithium-induced change in the sodium excretion in a dose-dependent manner. Consistent with this finding, the plasma sodium level was significantly reduced in the LiCl-treated rats and normalized after treatment with TAM (**Figure 2D**). Furthermore, administration of TAM prevented the lithium-induced potassium excretion and reduced plasma levels of potassium at day 14 (**Table 1**). Treatment with TAM also reduced plasma lithium levels (**Table 1**).

**Table 1:** Functional data after lithium and TAM treatment.

	Control	LiCl	LiCl + TAM 25	LiCl + TAM 50
**DAY 7**				
Urinary potassium excretion (mmol/24 h/100 g)	1.37 ± 0.13	1.77 ± 0.08^∗^	1.82 ± 0.06^∗^	1.67 ± 0.09
**DAY 14**				
Plasma lithium (mmol/L)		0.9 ± 0.09	0.5 ± 0.08^#^	0.5 ± 0.07^#^
Plasma potassium (mmol/L)	4.59 ± 0.11	4.65 ± 0.19	3.95 ± 0.12^∗#^	4.0 ± 0.07^∗#^
Urinary potassium excretion (mmol/24 h/100 g)	1.19 ± 0.06	1.6 ± 0.13^∗^	1.14 ± 0.11^#^	0.93 ± 0.07^#^

*Value are mean ± SEM. Multiple comparisons between experimental groups were performed using a one-way ANOVA followed by using Tukey’s multiple-comparison test. P < 0.05 was considered statistically significant indicated by ^∗^P < 0.05 compared to control and ^#^P < 0.05 compared to LiCl. LiCl, Lithium chloride, TAM 25, Tamoxifen 25 mg/kg, TAM 50, Tamoxifen 50 mg/kg.*

### TAM Affects the Expression of ENaC Subunits in Lithium-Treated Rats

**FIGURE 3:**
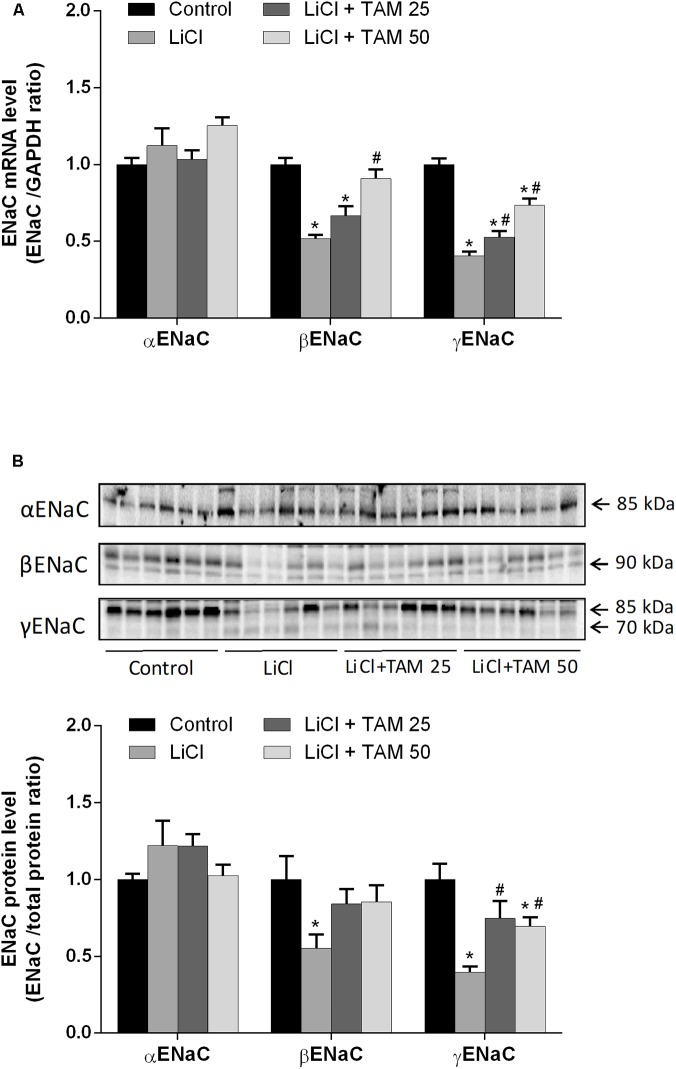
TAM affected the cortical expression of ENaC in the lithium-treated rats. **(A)** qPCR of the ENaC subunits α-, β-, and γ in cortical tissue. Data are normalized to those of GAPDH. **(B)** Western blot analysis performed for cortical αENaC, βENaC, and γENaC. Data are presented as the relative ratio of each protein normalized to total protein. Multiple comparisons between experimental groups were performed using one-way ANOVA followed by using Tukey’s multiple-comparisons test. *P* < 0.05 was considered statistically significant indicated by ^∗^*P* < 0.05 compared to controls and ^#^*P* < 0.05 compared to LiCl-treated rats. TAM, tamoxifen; LiCl, lithium chloride; ENaC, epithelial sodium channel; qPCR, quantitative polymerase chain reaction.

To investigate the effect of TAM on the expression of ENaC subunits, we examined cortical and OM tissues, the major sites of sodium reabsorption in the collecting duct. Expression of αENaC was unchanged at both the mRNA and protein levels after treatment with lithium alone or in a combination with TAM compared to those in the controls (**Figures 3A,B**). Consistent with previous observations (Nielsen et al., 2003) the protein expression of the ENaC subunits β and γ significantly reduced in cases of lithium-induced NDI. We also found that lithium administration reduced β- and γENaC expression at the transcriptional level. Treatment with TAM attenuated the downregulation of both subunits at the protein as well as mRNA levels (**Figures 3A,B**).

**FIGURE 4:**
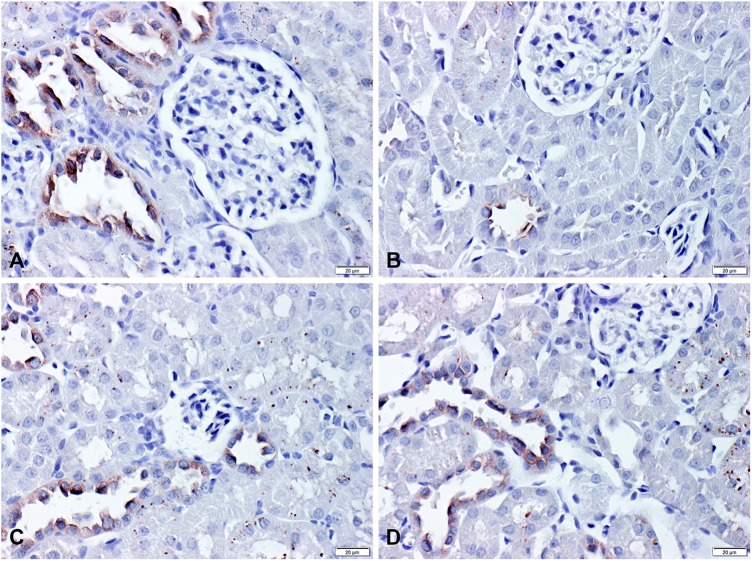
Localization of βENaC subunits in the lithium-treated rats. **(A–D)** Immunohistochemistry of βENaC in the apical domain of the cortical collecting ducts in tissues from control **(A)** and lithium-treated rats **(B)**. **(C,D)** LiCl-fed rats treated with TAM 25 mg/kg and TAM 50 mg/kg, respectively. Immunohistochemistry shows lesser labeling of βENaC in the lithium-treated rats compared to that in the controls. After TAM administration, stronger labeling of βENaC was detected in the lithium-treated rats, almost at the same level as that in the control rats. Magnification, ×40. TAM, tamoxifen; LiCl, lithium chloride; ENaC, epithelial sodium channel.

**FIGURE 5:**
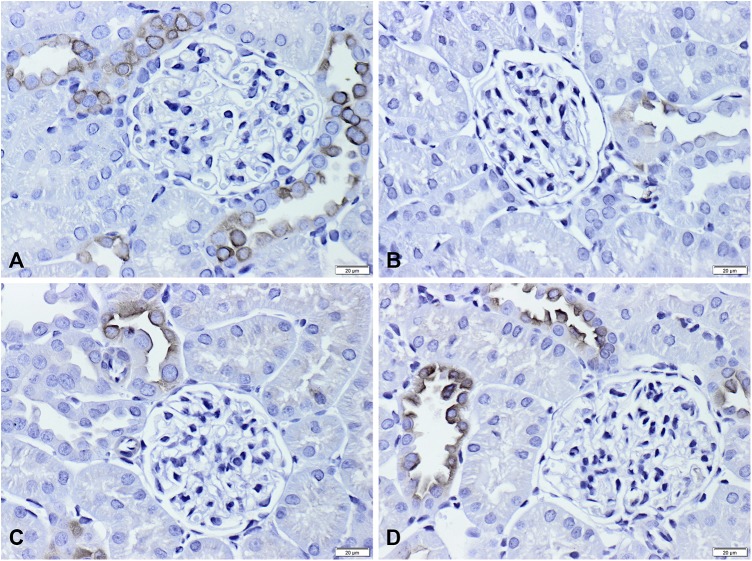
Localization of γENaC subunits in the lithium-treated rats. **(A–D)** Immunohistochemical microscopy of γENaC in the apical domain of the collecting duct in the cortical tissue from control **(A)** and LiCl-fed rats **(B)**. **(C,D)** LiCl-fed rats treated with TAM 25 mg/kg and TAM 50 mg/kg, respectively. Immunohistochemistry shows lesser labeling of γENaC in the lithium-treated rats compared to that in the control rats. After treatment with TAM, stronger labeling of γENaC was detected, almost at the same level as that in the control rats. Magnification, ×40. TAM, tamoxifen; LiCl, lithium chloride; ENaC, epithelial sodium channel.

These findings were further supported by those of IHC (**Figures 4**, **5**). Immunoperoxidase microscopy demonstrated a weak staining intensity of βENaC in the apical domain of the collecting duct cells in the LiCl-treated group compared to that in the control group (**Figures 4A,B**). Similarly, reduced labeling intensity was observed for γENaC in the LiCl group (**Figure 5B**) compared to that in the control group (**Figure 5A**). In the lithium-fed rats treated with TAM, the reduction in the labeling intensity of both ENaC subunits was less than that in the lithium-treated rats (**Figures 4C,D**, **5C,D** vs. **Figures 4B**, **5B**). These results suggest that TAM attenuated the downregulation of ENaC subunits, thereby decreasing the lithium-induced natriuresis in rats.

### Expression of Major Sodium Transporters in Response to Lithium and TAM Treatment

**FIGURE 6:**
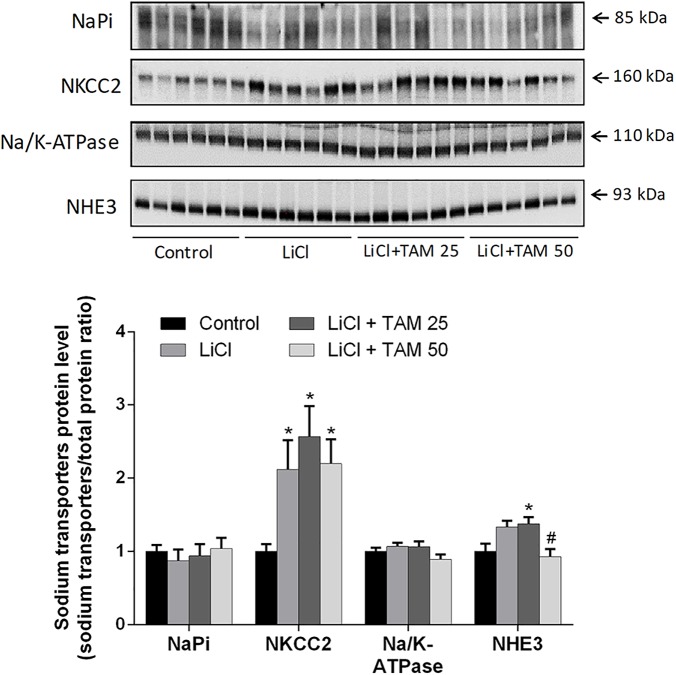
Expression of major sodium transporters in the lithium- and TAM-treated rats. Western blot analysis of cortical NaPi-2, NKCC2, Na/K-ATPase, and NHE3. Data are presented as the relative ratio of each protein normalized to total protein. Each bar represents the mean ± SEM values; *n* = 8 for control, *n* = 10 for LiCl, *n* = 10 for LiCl + TAM 25, and *n* = 12 for LiCl + TAM 50 groups. Multiple comparisons between the experimental groups were performed using one-way ANOVA followed by Tukey’s multiple-comparisons test. *P* < 0.05 was considered statistically significant indicated by ^∗^*P* < 0.05 compared to controls and ^#^*P* < 0.05 compared to LiCl. TAM, tamoxifen; LiCl, lithium chloride; ENaC, epithelial sodium channel.

We examined the effects of lithium and TAM on the expression of major renal sodium transporters located proximal to the ENaC expression sites in the cortex and OM. Semiquantitative immunoblotting was performed using antibodies to NaPi-2, NKCC2, Na-K-ATPase, and NHE3 (**Figure 6**). We did not find any change in the protein expression of NaPi-2 in the cortex and OM after lithium treatment. In addition, no effect of TAM was observed (**Figure 6**). NKCC2 is a cotransporter in the thick ascending limb (TAL), and the cortical expression of NKCC2 significantly increased after treatment with lithium as well as when it was administered in combination with TAM (**Figure 6**). Na-K-ATPase is found in all renal tubules segments. After 2 weeks of treatment with lithium, no changes were observed in Na-K-ATPase protein levels in the cortical and OM tissues. In addition, compared to lithium, TAM had no impact on the expression of Na-K-ATPase. NHE3 significantly increased after lithium treatment compared to that in the controls, and TAM treatment at a dose of 50 mg/kg prevented an increase in the abundance of NHE3 (**Figure 6**).

## Discussion

The main results of this study demonstrated that administration of TAM, a SERM, ameliorated lithium-induced natriuresis and kaliuresis by reducing urinary sodium and potassium excretion. Consistent with the finding, we demonstrated that TAM treatment attenuated the decrease in the protein and mRNA level expression of βENaC and γENaC in the cortical and OM collecting ducts in the lithium-treated rats, which was likely to play a role in the diminished renal tubular sodium excretion after TAM administration. An important outcome of this study was that we tested the efficacy of TAM when administrated after induction of lithium-induced NDI. The results indicated that TAM reduced the adverse effects of lithium-induced natriuresis, indicating that TAM might represent a new therapeutic approach for patients with lithium-induced NDI.

TAM is a SERM, which exerts its effect via binding to either the membrane-bound ERGP receptor or one of the nuclear ER receptors (ERα or ERβ) (Revankar et al., 2005; Catalano et al., 2014). We have recently demonstrated the expression of all three ER receptor subtypes in the kidney collecting ducts of rats with lithium-induced NDI (Tingskov et al., 2018). TAM can have either anti-estrogenic or estrogenic effects, depending on the type of tissues and cells (Lonard and Smith, 2002), and estrogen could induce renal tubular sodium reabsorption (Brunette and Leclerc, 2001). However, it is not clear whether TAM treatment had an effect on lithium-induced natriuresis.

Interestingly, we found that TAM treatment significantly attenuated the decrease in the expression of βENaC and γENaC subunits in the cortical and OM collecting ducts in the lithium-treated rats, which was likely to play a role in the diminished natriuresis after TAM administration. It has previously been demonstrated that downregulation of βENaC and γENaC might be the key molecular basis for the natriuresis associated with lithium-induced NDI (Nielsen et al., 2003). In addition, a study demonstrated that deficiency of βENaC or γENaC in mice resulted in high urinary sodium excretion and death before adulthood, indicating that ENaC is important for sodium reabsorption (McDonald et al., 1999).

Several studies have demonstrated a link between sex hormones and the regulation of renal sodium transporters/channels (Brunette and Leclerc, 2001; Jablonski et al., 2003; Gambling et al., 2004). Particularly, estrogen regulates the expression of ENaC subunits in the rat kidney (Gambling et al., 2004), and a recent study has shown that estrogen increases the apical membrane expression of the γENaC subunit in cortical collecting duct cells, which was dependent on PKCδ activation (Yusef et al., 2014). Consistent with these findings, it was reported that estrogen activated the amiloride-sensitive Na^+^ current (INa), which was attenuated by pretreatment of the cortical collecting duct cells with an inhibitor of PKCδ, matrix metalloproteinase, epidermal grow factor receptor, or PLC (Yusef et al., 2014). The findings indicated that estrogen could also regulate ENaC rapidly, independent of the non-genomic effects of aldosterone (Yusef et al., 2014).

In contrast, the underlying mechanisms by which TAM regulates the long-term protein expression of βENaC and γENaC are unclear. However, one could speculate that vasopressin might play a role as it has previously been shown that infusion of dDAVP (a vasopressin V2 receptor agonist) to Brattleboro rats lacking vasopressin induced a marked increase in β- and γENaC expression while no change was observed in the expression of the α-subunit (Nicco et al., 2001). Furthermore, several studies have demonstrated that estrogen receptors are expressed in the osmoreceptive areas of the brain that control vasopressin secretion and thereby give rise to circulating vasopressin levels (Sladek and Somponpun, 2008). Therefore, it is possible that TAM might stimulate vasopressin secretion leading to increased expression of the βENaC and γENaC subunits, which then ameliorates lithium-induced natriuresis.

We have also investigated the expression of other major sodium transporters including NaPi-2, NKCC2, Na-K-ATPase, and NHE3. The expression of these transporters increased or was unchanged after treatment with lithium, which indicates that the expression of these transporters is probably not involved in the increased urinary sodium excretion. These data are consistent with those of previous studies by Kwon et al. (2000), which demonstrated increased expression of NKCC2 and NHE3 in the lithium-treated rats. TAM had no impact on the regulation of NaPi-2, NKCC2, and Na-K-ATPase, in combination with lithium. In contrast, high-dose TAM treatment normalized the expression of NHE3 to the control levels in the lithium-treated rats. A previous study demonstrated that estrogen played a role in the regulation of NHE3 in the kidney cortex tissue from diabetic ovariectomized rats (Riazi et al., 2006), indicating that modulation of the estrogen receptor could play a role in the regulation NHE3.

This study showed that rats treated with lithium developed natriuresis even though the expression of the sodium transporters expressed in the proximal tubule, TAL, and distal convoluted tubule increased or was unchanged. ENaC finely tunes urinary sodium excretion and is the final route for sodium reabsorption along the nephrons, and therefore, the changes in ENaC regulation could be responsible for the natriuresis observed in the lithium-treated rats (Shalmi et al., 1998; Kwon et al., 2000). Lithium is known to substitute for sodium ion on sodium transporters; therefore, it could be argued that these main sodium transporters might provide a pathway for lithium entry into the cells since expression of ENaC subunits decreased in the lithium-fed rats.

Our study showed that TAM treatment was associated with reduced blood lithium levels; hence, we cannot exclude the probability of an interaction between TAM and lithium, which resulted in the reduced blood lithium levels (Tingskov et al., 2018). Since lithium-induced polyuria in animals correlated with plasma lithium levels (Marples et al., 1995), we may postulate that urinary sodium excretion could also be affected by the plasma lithium levels. Although the lithium levels in our study were within the therapeutic plasma concentration range (0.5–1.2 mM) (Klein and Melton, 1996; Wada, 2009); we cannot exclude the possibility that the TAM-mediated effects on the kidney were, at least in part, acted by the lower levels of plasma lithium.

## Conclusion

We have shown that TAM treatment attenuated the increased urinary sodium excretion, even after the establishment of natriuresis in the lithium-treated rats. Future studies are warranted to investigate whether the effects of TAM are sustained and if TAM could be a useful therapeutic approach for patients with lithium-induced NDI.

## Author Contributions

SJT, T-HK, JF, and RN designed experiments. SJT performed experiments. SJT and RN analyzed experimental results. SJT and RN wrote the manuscript. SJT, T-HK, JF, and RN finalized and approved the final version of the manuscript.

## Conflict of Interest Statement

The authors declare that the research was conducted in the absence of any commercial or financial relationships that could be construed as a potential conflict of interest.
